# The role of S100B/RAGE-enhanced ADAM17 activation in endothelial glycocalyx shedding after traumatic brain injury

**DOI:** 10.1186/s12974-022-02412-2

**Published:** 2022-02-11

**Authors:** Zhimin Zou, Li Li, Qin Li, Peng Zhao, Kun Zhang, Chengyong Liu, Daozhang Cai, Marc Maegele, Zhengtao Gu, Qiaobing Huang

**Affiliations:** 1 Guangdong Provincial Key Lab of Shock and Microcirculation, Department of Pathophysiology, School of Basic Medical Sciences, Southern Medical University, Guangzhou, 510515 Guangdong China; 2 Department of Treatment Center for Traumatic Injuries, The Third Affiliated Hospital of Southern Medical University, Guangzhou, 510630 Guangdong China; 3 Academy of Orthopedics of Guangdong Province, Orthopedic Hospital of Guangdong Province, Guangdong Provincial Key Laboratory of Bone and Joint Degenerative Diseases, The Third Affiliated Hospital of Southern Medical University, Guangzhou, 510630 Guangdong China; 4 Center of TCM Preventive Treatment, Integrated Hospital of Traditional Chinese Medicine, Southern Medical University, Guangzhou, 510315 Guangdong China; 5 Department of Orthopedics, Center for Orthopaedic Surgery, The Third Affiliated Hospital of Southern Medical University, Academy of Orthopedics Guangdong Province, Guangzhou, 510630 Guangdong Germany; 6 Institute for Research in Operative Medicine (IFOM), University Witten/Herdecke (UW/H), Campus Cologne-Merheim, Ostmerheimerstr. 200, 51109 Köln, Germany; 7 Department for Trauma and Orthopedic Surgery, Cologne-Merheim Medical Center (CMMC), University Witten/Herdecke (UW/H), Campus Cologne-Merheim, Ostmerheimerstr. 200, Köln, 51109 China

**Keywords:** Traumatic brain injury, Secondary injury, Blood–brain barrier, Endothelial glycocalyx, S100B/RAGE, ADAM17

## Abstract

**Background:**

Traumatic brain injury (TBI) remains one of the main causes for disability and death worldwide. While the primary mechanical injury cannot be avoided, the prevention of secondary injury is the focus of TBI research. Present study aimed to elucidate the effects and mechanisms of S100B and its receptor RAGE on mediating secondary injury after TBI.

**Methods:**

This study established TBI animal model by fluid percussion injury in rats, cell model by stretch-injured in astrocytes, and endothelial injury model with conditioned medium stimulation. Pharmacological intervention was applied to interfere the activities of S100B/RAGE/ADAM17 signaling pathway, respectively. The expressions or contents of S100B, RAGE, syndecan-1 and ADAM17 in brain and serum, as well as in cultured cells and medium, were detected by western blot. The distribution of relative molecules was observed with immunofluorescence.

**Results:**

We found that TBI could activate the release of S100B, mostly from astrocytes, and S100B and RAGE could mutually regulate their expression and activation. Most importantly, present study revealed an obvious increase of syndecan-1 in rat serum or in endothelial cultured medium after injury, and a significant decrease in tissue and in cultured endothelial cells, indicating TBI-induced shedding of endothelial glycocalyx. The data further proved that the activation of S100B/RAGE signaling could promote the shedding of endothelial glycocalyx by enhancing the expression, translocation and activity of ADAM17, an important sheddase, in endothelial cells. The damage of endothelial glycocalyx consequently aggravated blood brain barrier (BBB) dysfunction and systemic vascular hyper-permeability, overall resulting in secondary brain and lung injury.

**Conclusions:**

TBI triggers the activation of S100B/RAGE signal pathway. The regulation S100B/RAGE on ADAM17 expression, translocation and activation further promotes the shedding of endothelial glycocalyx, aggravates the dysfunction of BBB, and increases the vascular permeability, leading to secondary brain and lung injury. Present study may open a new corridor for the more in-depth understanding of the molecular processes responsible for cerebral and systemic vascular barrier impairment and secondary injury after TBI.

**Supplementary Information:**

The online version contains supplementary material available at 10.1186/s12974-022-02412-2.

## Introduction

Traumatic brain injury (TBI) remains one of the leading causes of death and disability worldwide with more than 10 million people hospitalized every year [1]. TBI is viewed as a cascade that involves the initial insult which triggers the primary injury and the secondary damage to the brain and non-nervous system on the following [2]. S100B, the most abundant calcium-binding protein in nerve tissue, especially in astrocytes, is one of damage-associated molecular patterns (DAMPs) released after early or primary brain injury and contributes to secondary injury [3]. Regarded as a TBI biomarker, S100B has been shown to correlate with injury magnitude, survival and neurologic outcome [4, 5]. Studies have shown that astrocyte activation and abnormal BBB function can lead to a significant increase of S100B in circulating serum and cerebrospinal fluid [6]. By binding with receptors for advance glycation endproducts (RAGE), S100B mediates a series of pathophysiological processes as an extracellular regulator for various cells, including endothelial cells, astrocytes, etc. [7–9].

The endothelial glycocalyx (EG) represents a layer of negatively charged, brush-like polysaccharide–protein complex structures, which is regarded as a protective barrier on top of endothelial cells in blood vessels [2, 10]. EG is highly vulnerable in the early stage of vascular endothelial dysfunction, it’s damage is believed to lead to traumatic endotheliopathy. Various harmful components, including inflammatory factors and sheddases, may affect the stability of EG in TBI [11]. Studies have reported that the systemic breakdown of the glycocalyx can be observed as early as at 15 min after the occurrence of TBI combined with hemorrhagic shock in vivo [12]. Simultaneously, increased plasma levels of S100B in trauma patients have been related to endothelial cell damage [13], but the underlying mechanisms remain yet to be elucidated.

Among many possible mechanisms of EG injury, the sheddases have attracted the most attention over the past years. It is found that a sheddase, A disintegrin and metalloprotease 17 (ADAM17), also known as tumor necrosis factor (TNF)-alpha converting enzyme (TACE), was significantly increased in the early stages of brain injury [14, 15]. Recent studies have suggested that S100 family proteins affect tumor metastasis and development by up-/down-regulating the expression and activity of matrix metalloproteinases [16]. However, the effects of S100B and ADAM17 in the breakdown of vascular glycocalyx in TBI are still not fully described; and whether S100B/RAGE signal can regulate ADAM17 expression and activity in TBI, thus contributing to secondary brain injury remains to be clarified.

In the present study, using lateral fluid percussion brain injury rat model and stretch injury astrocyte model with pharmacological intervention, we elucidated that there was mutual enhancing regulation between S100B/RAGE and the activation of this signaling pathway was involved in ADAM17-mediated endothelial glycocalyx shedding, blood–brain barrier dysfunction and secondary damage after TBI.

## Methods

**Fig. 1:**
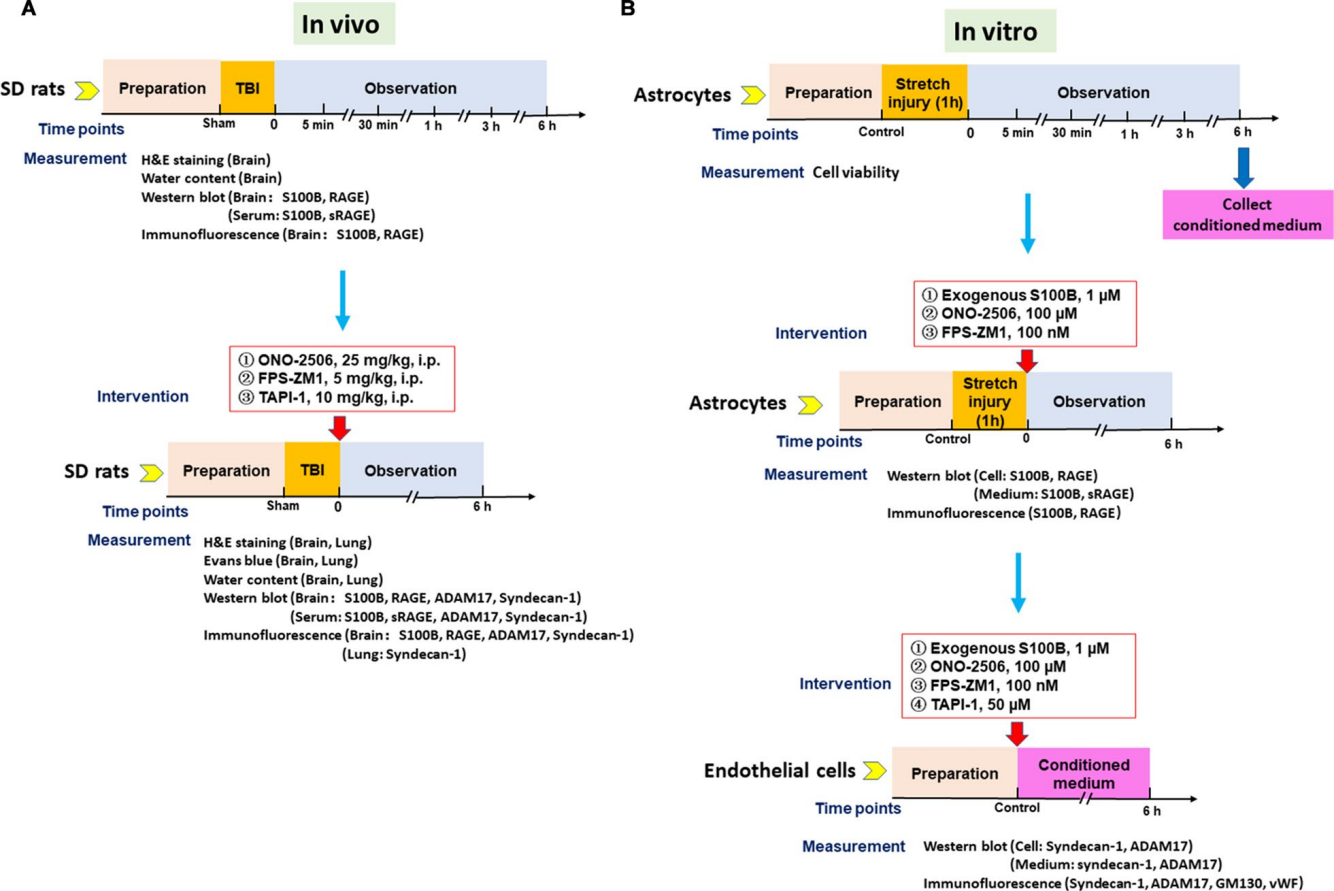
Flow chart of the in vivo*/*in vitro experimental protocols.** A** Protocol of lateral fluid percussion brain injury rat model. **B** Protocol of stretch injury astrocyte model

The lateral fluid percussion brain injury rat was applied as in vivo TBI model and the protocol is shown in Fig. 1a and the details of the animal number used in this study is shown in Table 1. The in vitro TBI cellular models were produced using cultured astrocytes treated with stretch injury and cultured primary rat aortic endothelial cells (RAECs) treated with conditioned medium of injured astrocytes. The protocol is shown in Fig. 1b.

**Table 1:** Number of animals in different experimental groups for various parameter measurement

Group assessment	Mortality rate	Brain /lung water content	H&E staining	Immuno-fluorescence	Western blot	Evans blue	Total
Sham	9	6	3	3	(6)	6	27
5 min after TBI	9	6	3	3	(6)	0	21
30 min after TBI	9	6	3	3	(6)	0	21
1 h after TBI	9	6	3	3	(6)	0	21
3 h after TBI	9	6	3	3	(6)	0	21
6 h after TBI	9	6	3	3	(6)	6	27
TBI + ONO-2506	9	6	3	3	(6)	6	27
TBI + FPS-ZM1	9	6	3	3	(6)	6	27
TBI + TAPI-1	9	6	3	3	(6)	6	27
Total	90	54	27	27		30	228

Number in parentheses means the same rats were used for assessment of mortality rate

### The in vivo TBI model of rats

#### Animals

The animals were purchased from the Experimental Animal Center of the Southern Medical University in China (Certification number: SYXK (Yue) 2016–0167). This study was approved by the Animal Care and Use Committee of the Southern Medical University, China (Approval number: L2019134). The experimental procedure followed the United States National Institutes of Health Guide for the Care and Use of Laboratory Animals (NIH Publication No 85–23, revised 1985). Two hundred and twenty-eight specific-pathogen-free male Sprague-Dawley rats aged 12 weeks and weighing 250–300 g were housed individually under controlled environment with a 12-h light/dark cycle and unrestricted access to pellet food and water. Rats were randomly assigned to different experimental groups. All surgical interventions were performed under anesthesia with a mixture of 13.3% urethane and 0.5% chloralose (0.65 mL/100 g body weight, intraperitoneally). All efforts were made to reduce the number of animals used and to minimize animal discomfort.

#### Lateral fluid percussion brain injury

The TBI animal model was produced by lateral fluid percussion with surgical procedures performed as previously described [17]. Briefly, anesthetized rats were placed in a stereotaxic frame. After incision of the scalp, the temporal muscles were separated and a 4.8 mm craniotomy was drilled (2.5 mm lateral to the sagittal sinus and centered between bregma and lambda). A hollow female Luer-Lok fitting was placed directly over the dura and rigidly fixed using dental cement. Before the induction of trauma, the female Luer-Lok was connected to the fluid percussion injury device via a transducer (Biomedical Engineering Facility, Medical College of Virginia, USA). For the infliction of TBI, a metal pendulum was released from a pre-selected height, leading to a rapid injection of normal saline into the closed cranial cavity. The pulse of increased intracranial pressure of 21–23 ms duration was controlled and recorded by an oscilloscope (Agilent 54622D, MEGAZoom, Germany). The severity of injury inflicted on the animal was altered by adjusting the amount of pressure generated by the pendulum. For the present experiment, a severe injury level was induced (3.5 ± 0.2 atm). The sham group animals underwent identical preparatory procedures, including craniotomy, but were not was not inflicted with TBI. Rats were sacrificed with pentobarbital (50 μg/kg i.p.) at 5 min, 30 min, 1 h, 3 h and 6 h. Blood samples were collected by cardiac puncture, then serum was obtained by centrifuging the blood at 3000 r/min. The brain, and lungs were carefully collected at the same time. The general pathological changes of the brain were observed by detection of brain histology and water content at 5 min, 30 min, 1 h, 3 h and 6 h following TBI. The expression of S100B and RAGE in brain tissue, the levels of S100B, sRAGE in serum were measured in sham and TBI group at 5 min, 30 min, 1 h, 3 h and 6 h following TBI.

#### Pharmacological intervention in rats

Rats were randomly assigned to different experimental groups. To clarify the effects and signaling relationship of S100B, RAGE, ADAM17 on the development of TBI and the secondary injury of brain and lungs, rats were randomized into sham group, TBI group, TBI + ONO-2506 (S100B chemical inhibitor) group, TBI + FPS-ZM1 (RAGE chemical inhibitor) group, and TBI + TAPI-1 (TNF-alpha processing inhibitor-1, an ADAM17 inhibitor) group. ONO-2506 [18] (25 mg/kg; GLPBIO GC15105, USA), FPS-ZM1 [19] (5 mg/kg; GLPBIO GC10652, USA), or TAPI-1 [20] (10 mg/kg; GLPBIO GC12344, USA) was, respectively, applied through intraperitoneal injection immediately after the onset of TBI, and the relevant parameters were measured 6 h after the onset of TBI.

#### Histopathological analysis

The cortex of injured-side brain and the lungs from sham and TBI rats were quickly excised, sliced into transverse or longitudinal sections, and fixed in 10% neutral-buffered formalin. The tissues were then embedded in paraffin blocks, and serial sections were stained with hematoxylin and eosin (H&E) for microscopic evaluation at 50, 100 or 200× magnification. Morphological changes were blindly assessed and graded by two certified pathologists using the injury score developed.

#### Measurement of tissue water content

The rats were sacrificed at 5 min, 30 min, 1 h, 3 h and 6 h following TBI, the whole brain and lung were excised and weighed. Then, the tissues were placed in an oven at 80 °C loosely wrapped in filter paper for 72 h to achieve dry weight, and the water content of tissues was calculated with ratio of (wet weight–dry weight)/wet weight to assess brain edema.

#### Evans blue extravasation

BBB permeability and pulmonary microvascular permeability was evaluated by measuring the extravasation of Evans blue (EB) dye (Sigma Aldrich). The EB dye (2%, 4 mL/kg) was injected intravenously 2 h prior to the sacrificing of rats. Following euthanasia, rats were transcardially perfused with phosphate buffered saline (PBS) through the left ventricle of the heart to sufficiently eliminate the intravascular-localized dye. The brain and lung were removed. Each sample was immediately weighed and homogenized in 1 mL of 50% trichloroacetic acid solution. The homogenate was centrifuged (12,000×*g*, 20 min), and the supernatant was transferred to a new tube and diluted 1:3 with ethanol. Its absorbance was determined at 610 nm using a spectrophotometer (Thermo Fisher Scientific, China). A standard curve was used to calculate the quantity of dye, which was expressed as micrograms per gram of brain and lung tissue.

### The astrocyte stretch injury model

The astrocyte stretch injury model was applied to reproduce the in vitro injury process of nerve cells after TBI [21].

#### Animals

Twenty specific-pathogen-free male Sprague-Dawley rat pups aged 1–4 days used for astrocyte isolation were purchased from the Experimental Animal Center of the Southern Medical University in China (Certification number: SYXK (Yue) 2016–0167). This study was approved by the Animal Care and Use Committee of the Southern Medical University, China (Approval number: L2019134). The experimental procedure followed the United States National Institutes of Health Guide for the Care and Use of Laboratory Animals (NIH Publication No 85–23, revised 1985).

#### Culture of primary astrocytes

The protocol of primary astrocyte culture was similar to that described previously [21]. Cortices from 1 to 4-day-old rat pups were used for astrocyte isolation. After decapitation of the rats and removal of the meninges, the cortices were digested by 0.25% Trypsin, and the cells were treated with DNase I (Sigma) before centrifugation at 1000×*g* for 3 min. Cells were cultured on poly-l-lysine-coated dishes at a density of 1 × 10^6^ cells per 10-cm dish in minimum essential medium with 10% fatal bovine serum. The medium was changed to new medium on the following day. The medium was changed every 3 days, the density of cells can reach 80–90% at 7 days. Subsequently, the primary astrocytes were collected and identified with glial fibrillary acid protein (GFAP) expression through immunofluorescence to assess nerve cell purity. Next experiment would be carried out, while the astrocyte purity reached 99%.

#### The stretch injury of astrocytes

The protocol of astrocyte stretch injury model was similar to those described previously [22]. Equiaxial stretch (20% strain, 1.0 Hz frequency) was applied to cultured astrocytes for 1 h, by a Flexcell^®^ FX-5000™ Tension System (Flexcell, USA) with specific designed 6-well plates (BioFLEX^®^). The culture media of astrocytes in stretch injury group and control group were collected at this time and will be used as conditioned medium. Again, to clarify the effects and signaling relationship of S100B, RAGE, ADAM17 on stretch injury, ONO-2506 (100 µM) [18], FPS-ZM1 (100 nM) [23] or TAPI-1 (50 µM) [24] were, respectively, applied for 6 h after stretch injury. To further verify the effect of S100B on astrocyte injury, exogenous S100B (1 µM) [8] was directly administrated to normal astrocytes for 6 h. The cell viability and relevant parameters were detected as indicated.

#### Detection of astrocyte viability by CCK-8

A cell counting kit (CCK-8 kit; Beyotime Biotechnology; C0038; China) was used to detect cell viability and cells were cultured in the relevant condition as above. Cells from different treatment groups were counted, adjusted the concentration to 1 × 10^5^ mL, and seeded in a 96-well plate with 100 μL/well. Cells were seeded in triplicate for each treatment group. The 96-well plate was placed in the incubator (37 °C and 5% CO_2_) and cells were cultured till the indicated time. 10 μL CCK-8 solution was added to each well and the cell culture plate was incubated for 1 h. The absorbance at 450 nm was detected using a plate reader. Blank wells (culture media and CCK) and control wells (untreated cells, culture media, and CCK) were also detected.

### The endothelial injured model

To reproduce the systemic secondary injury model of endothelial cells after TBI, aortic endothelial cells was stimulated with conditioned medium from the astrocyte stretch injury model.

#### Animals

Twenty specific-pathogen-free male Sprague-Dawley rats aged 12 weeks and weighing 250–300 g used endothelial cell isolation were purchased from the Experimental Animal Center of the Southern Medical University in China (Certification number: SYXK (Yue) 2016–0167). This study was approved by the Animal Care and Use Committee of the Southern Medical University, China (Approval number: L2019134). The experimental procedure followed the United States National Institutes of Health Guide for the Care and Use of Laboratory Animals (NIH Publication No 85–23, revised 1985). The animals were housed individually under controlled environment with a 12-h light/dark cycle and unrestricted access to pellet food and water. Rats were randomly assigned to different experimental groups. All surgical interventions were performed under anesthesia with a mixture of 13.3% urethane and 0.5% chloralose (0.65 mL/100 g body weight, intraperitoneally). All efforts were made to reduce the number of animals used and to minimize animal discomfort.

#### The culture of primary rat aortic endothelial cells

A TBI-induced endothelial injured model was produced using primary rat aortic endothelial cells (RAECs) stimulated with conditioned medium from the astrocyte stretch injury model. The protocol for the isolation and identification of RAECs were modified from Zhu et al. [25]. The animals were first anesthetized and disinfected for the exposure of the aorta. The aorta was quickly isolated after flushing the blood through the left ventricle with PBS and dissected to remove adipose tissue and small colateral vessels. The aortic tissues were then cut into 2 to 5 mm long rings and each aortic ring was cut open and immediately placed with the lumen side down into a six‐well plate containing up to 50 µl of endothelial cell growth medium with endothelial cell growth factor, fetal bovine serum, penicillin, and streptomycin (Cat #1001; ScienCell, Carlsbad, CA). When the sprouting and cell growth reached 80% confluency, the aortic segments were gently removed, the cells were harvested and the von Willebrand factor (vWF) expression was detected through immunofluorescence to identify endothelial cells and to assess purity.

#### The treatment of RAECs with conditioned medium from injured astrocytes

The above-mentioned conditioned medium from astrocyte stretch injury model was applied to RAECs for 6 h, with supplement of vascular endothelial cell growth factor (Cat #1052; ScienCell, Carlsbad, CA). To further verify the effect of S100B on endothelial cells, exogenous S100B (1 µM) (GuangZhou BlueLife Biotechnology Co., Ltd) was directly used to stimulate normal RAECs. To clarify the effects and the signaling relationship of S100B, RAGE, and ADAM17 on glycocalyx shedding of endothelial cells, ONO-2506 (100 µM), FPS-ZM1 (100 nM) or TAPI-1 (1 µM) were added for 6 h while incubating with conditioned medium. The relevant parameters were detected as indicated. The experimental protocol in endothelial injury model is shown the lower right panel of the flow chart in Fig. 1.

### General detection

#### Antibodies

The following primary antibodies were used for western blotting or immunofluorescence: GAPDH (AF7021; Affinity), S100B (ab52642; Abcam), RAGE and sRAGE (ab216329; Abcam), GFAP (ab7260; Abcam), vWF (ab6994, Abcam), syndecan-1 (ab128936; Abcam), ADAM17 (ab39162, Abcam), GM130 (DF7556; Affinity). The following secondary antibodies were used: peroxidase-conjugated goat anti-rabbit, Alexa Fluor 488- and Alexa Fluor 594-conjugated secondary antibody were bought from the Beijing Ray Antibody Biotech Company.

#### Western blotting

Samples were separated by SDS-PAGE under reducing conditions and transferred onto polyvinylidene difluoride (PVDF) membranes (Immobilon-P, Millipore). Membranes were blocked with 5% BSA in TBS (50 mM Tris, 150 mM NaCl, pH 7.4), and subsequently incubated with primary antibodies in 0.1% Tween-TBS with 1% BSA and washed in 0.1% Tween-TBS. Bound primary antibodies were detected with peroxidase-conjugated goat anti-rabbit antibodies using the ECL detection system Super Signal West Pico (Thermo Fisher Scientific, Germany).

#### Immunofluorescence

After fixation, dehydration, the frozen sections of SD rat brain and lung tissues were prepared. Cells were grown on a confocaldish and treated as indicated. The tissue sections and cells were fixed with 4% methanol, perforation with 0.1% Triton x-100 and blocked for 1 h in 5% BSA and 0.1% Tween-20 in PBS at room temperature. The primary antibodies were applied to the sample, followed by Alexa Fluor 488- and Alexa Fluor 594-conjugated secondary antibody. The images were obtained by laser scanning confocal microscopy (LSCM; Carl Zeiss Microscopy/Zeiss LSM780; Germany). The immunofluorescence intensities of target proteins in brain tissue or cultured cells from five fields per sample in each group were quantified and reported as relative fluorescence units (RFUs).

### Statistical analysis

Statistical analyses were carried out using GraphPad Prism 6.0 (San Diego, USA). Data are from at least three independent experiments performed in duplicate and are expressed as mean ± SD. Statistical comparisons of the results were performed using one-way analysis of variance (ANOVA). A* p* < 0.05 was considered to be statistically significant.

## Results

### TBI was accompanied with activation of S100B/RAGE signaling

#### Characteristics of the experimental TBI animal model

**Fig. 2:**
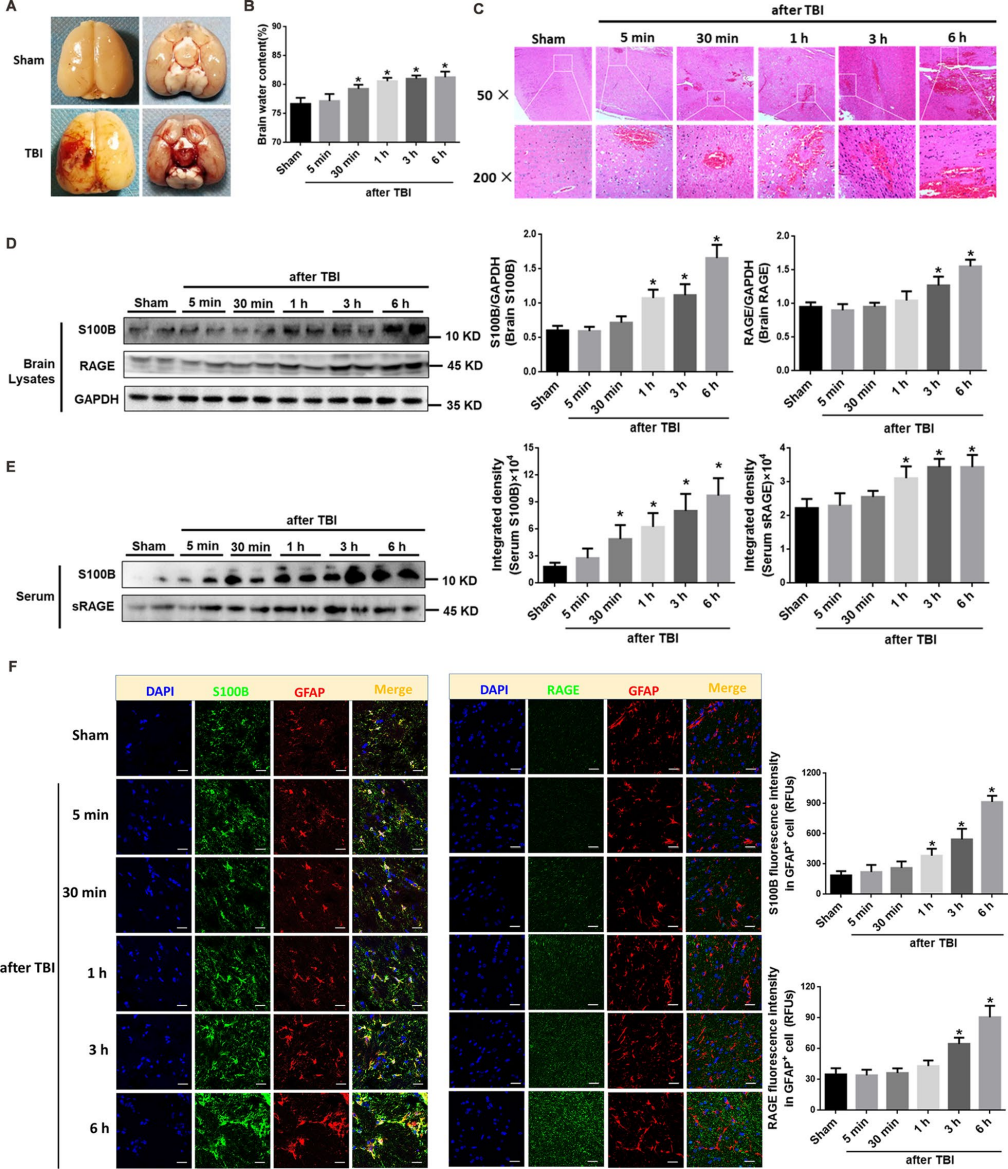
Injury of brain was accompanied with activation of S100B/RAGE signal and endothelial glycocalyx shedding after TBI. **A** Gross observation of brain tissue in TBI rats. TBI was achieved by lateral fluid percussion brain injury.** B** Quantification of water content of brain at different timepoints after TBI calculated with ratio of (wet weight − dry weight)/wet weight. *p < 0.05 compared with Sham group, *n* = 6. **C** Representative H&E staining images showing the histology of cortex of injured-side brain tissue at different timepoints after TBI. **D** Representative blots of S100B, RAGE in tissue lysates of brain peri-injury cortex at different timepoints after the onset of TBI. GADPH was used as a soluble loading control (left panel). In addition, quantification (histograms of right panels) of S100B, RAGE from representative blots shown in left panel. *p < 0.05 compared with Sham group, *n* = 6. **E** Representative blots of S100B, sRAGE in the serum of TBI rats (left panel). In addition, quantification (histograms of right panels) of S100B, sRAGE contend in serum from representative blots shown in left panel, presenting in band intensity. *p < 0.05 compared with Sham group, *n* = 6. **F** Representative confocal images showing the subcellular localization of cytocolic S100B with GFAP and the existence of membrane RAGE in GFAP^+^ cells in the cortex of injured-side brain tissue. DAPI was used as a nuclear marker. Scale bar = 50 μm. The immunofluorescence intensities of S100B and RAGE in brain tissue from five fields per sample in each group were quantified and reported as relative fluorescence units (RFUs). *p < 0.05 compared with Sham group, *n* = 3

The onset of TBI was characterized by brain hyperemia, tissue contusion and swelling in the area of impact, and there was obvious hematocele in the ventral surface of the brain (Fig. 2A). The water content in the injured brain began to increase significantly in a time-dependent manner 30 min after TBI (Fig. 2B). H&E staining showed that the brain tissue in sham group were arranged uniformly and the intercellular space was normally distributed. Nerve cells were accompanied in abundant cytoplasm with deep-dyed big nucleolus. The structure of the brain tissues obtained from injured rats was damaged directly through the mechanical impact reflected by ruptured blood vessels and bleeding, resulting in subsequent diffuse hemorrhagic foci. Nuclei became condensed with deeper staining. With the expansion of the hemorrhagic foci, the nerve cells swelled and the cytoplasm vacuolated. Infiltration of inflammatory cells, or obvious leakage of red blood cells could be observed after experimental TBI and this pathological damage increased significantly with the prolonged observation times (Fig. 2C). There was certain mortality in this severe TBI rat model at different timepoints (5 min, 30 min, 1 h, 3 h, 6 h), respectively (Table 2).

**Table 2:** Mortality of rats after TBI

Group	*n*	No. death	Mortality (%)
Sham	9	0	0.00
5 min after TBI	9	2	22.22*
30 min after TBI	9	1	11.11*
1 h after TBI	9	2	22.22*
3 h after TBI	9	2	22.22*
6 h after TBI	9	3	33.33*
TBI + ONO-2506	9	0	0^#^
TBI + FPS-ZM1	9	0	0^#^
TBI + TAPI-1	9	1	11.11^#^

Mortality (%) = Death numbers/*n* × 100%

^*^p < 0.05 compared Sham group

^#^*p* < 0.05 compared TBI group (6 h after TBI)

#### Brain injury was accompanied by elevated S100B, RAGE

Compared with those in the relevant areas in sham group, the expression of S100B and RAGE in cerebral cortices at the edge of injury was increased significantly after TBI (Fig. 2D). Serum S100B and sRAGE levels also increased gradually and significantly after TBI (Fig. 2E). The level of sRAGE in serum was increased at 1 h after TBI, which was earlier as the increased expression of RAGE in the brain tissue (Fig. 2E). The results from immunofluorescence under confocal microscope of brain cerebral cortices were consistent with those from western blot. Precisely, the enhanced staining of cytosolic S100B was co-localized with GFAP-positive (GFAP^+^) cells and membrane RAGE was also increased in the area of GRAP^+^ cells at 6 h after TBI (Fig. 2F).

#### Astrocyte TBI model was also characterised by elevated S100B, RAGE

**Fig. 3:**
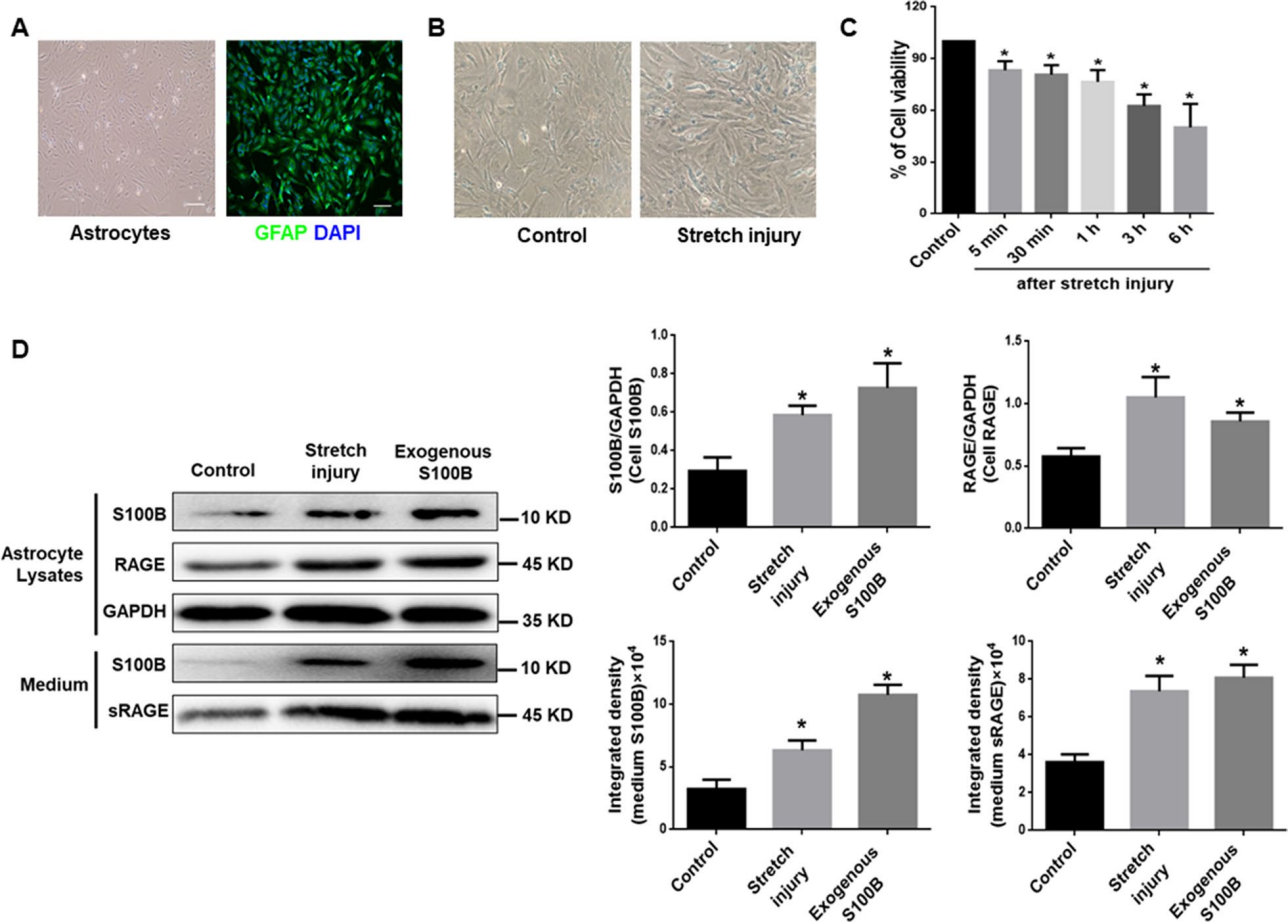
Astrocyte TBI model was characterised by elevated S100B, RAGE. **A** Morphology of primary cultured rat astrocytes identified with positive staining of GFAP. DAPI was used as a nuclear marker. Scale bar = 100 μm. **B** Morphological changes of astrocytes before and after the equiaxial stretch injury induced by Flexcell^®^ FX-5000™ Tension System, × 100. **C** Cell viability of astrocytes at different timepoints after stretch injury detected by CCK-8. *p < 0.05 compared with the Control group, *n* = 4. **D** Representative blots of S100B and RAGE in astrocytes, S100B and sRAGE in cultured medium after stretch injury or exogenous S100B administration. And quantification (histograms of right panels) of S100B, RAGE in cell lysates and S100B, sRAGE in medium from representative blots shown in left panel. GADPH was used as a soluble loading control for cellular protein content. The content of S100B and sRAGE in cultured medium was shown directly by gray scale. *p < 0.05 compared with Control group, *n* = 3

Cultured primary rat astrocytes were dispersed evenly with flat-spindle-shaped in “slab stone”-like arrangement under light microscope and were identified as GFAP^+^ cells (Fig. 3A). The cells presented with morphology change, and poor cell refraction after mechanically stimulated with stretching (Fig. 3B). Compared with control group, cell viability decreased in a time-dependent manner (Fig. 3C). Stretch injury induced the elevation of S100B in astrocytes and medium. Both stretch injury and exogenous S100B could enhanced the expression of RAGE in astrocytes and sRAGE in medium (Fig. 3D). These data in Figs. 2 and 3 suggest that TBI triggered the up-regulation of S100B/RAGE signal in the pathogenesis of primary and secondary injury.

### The mutual regulation of S100B and RAGE in TBI

#### Inhibition of S100B reduce RAGE expression and sRAGE secretion in animal and astrocyte TBI models

**Fig. 4:**
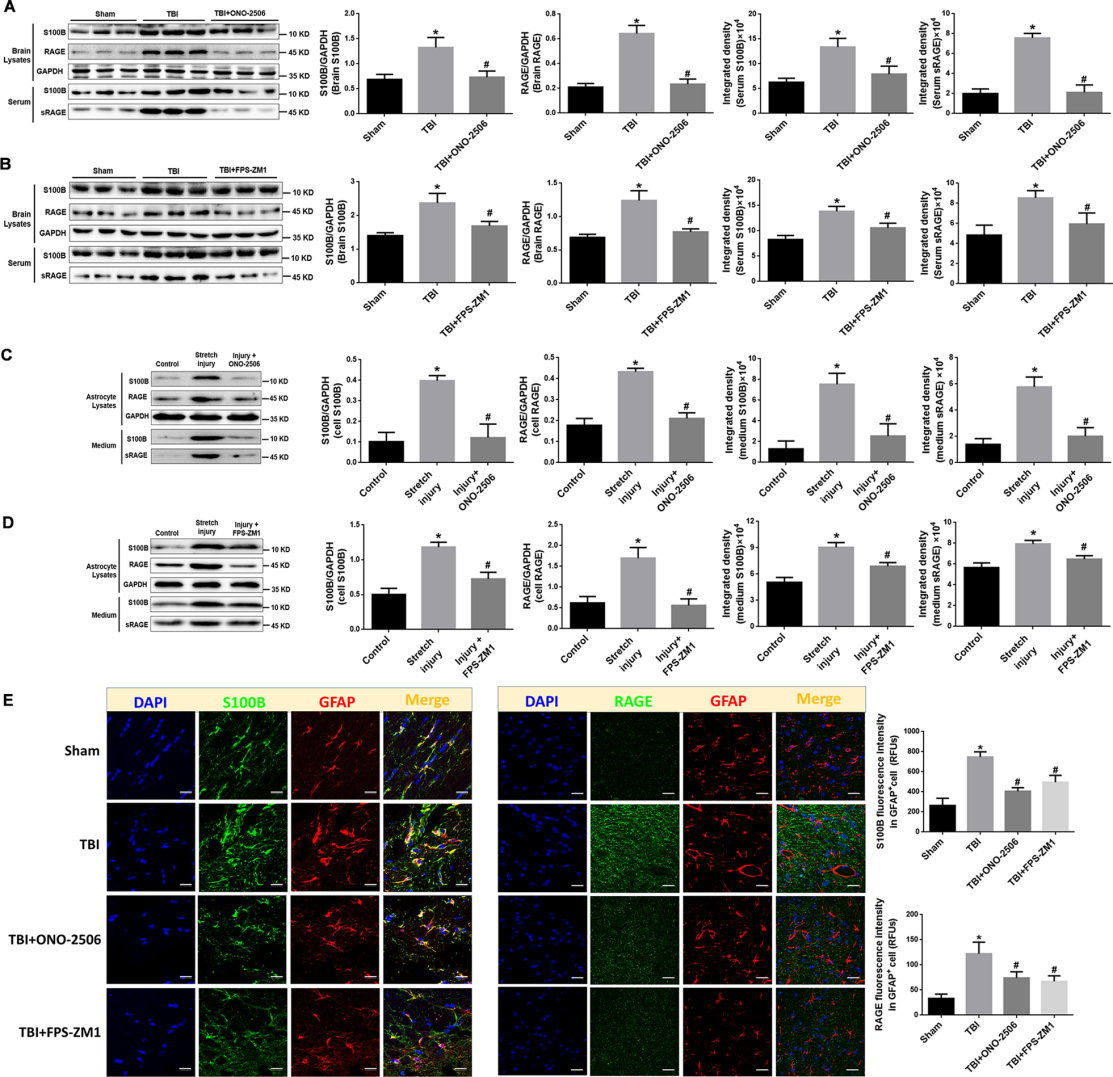
Inhibition of S100B/RAGE signal attenuated TBI-induced up-regulation of S100B/RAGE over-expression and sRAGE secretion in astrocyte and animal models. **A**, **B** Representative blots of S100B and RAGE in injured brain, S100B and sRAGE in serum of TBI rats, or TBI plus ONO-2506 (**A**) or FPS-ZM-1 (**B**). GADPH was used as a soluble loading control (left panel). In addition, quantification (histograms of right panels) of S100B, RAGE in brain lysates and S100B, sRAGE in serum from representative blots shown in left panel. *p < 0.05 compared with Sham group, ^#^*p* < 0.05 compared with TBI group, *n* = 6. **C**, **D** Representative blots of S100B and RAGE in astrocytes, S100B and sRAGE in cultured medium after stretch injury or injury plus S100B inhibitor ONO-2506 (**D**) or FPS-ZM-1 (**E**). GADPH was used as a soluble loading control (left panel). In addition, quantification (histograms of right panels) of S100B, RAGE in cell lysates and S100B, sRAGE in medium from representative blots shown in left panel. *p < 0.05 compared with Control group, ^#^*p* < 0.05 compared with Stretch injury group, *n* = 3. **E** Representative confocal images showing the subcellular localization of S100B with GFAP and the existence of RAGE in GFAP^+^ cells in the brain tissue of rats treated with TBI, TBI plus ONO-2506 or FPS-ZM-1. Scale bar = 50 μm. The immunofluorescence intensities of S100B and RAGE in brain tissue from five fields per sample in each group were quantified and reported as relative fluorescence units (RFUs). *p < 0.05 compared with Sham group, ^#^*p* < 0.05 compared with TBI group, *n* = 3

The administration of ONO-2506 in rat attenuated TBI-induced increase of RAGE expression in cerebral cortices and sRAGE secretion to the serum (Fig. 4A, E). Consistently, the administration of S100B inhibitor ONO-2506 abolished the stretch injury-induced increase of RAGE expression and sRAGE secretion in astrocytes (Fig. 4C).

#### Inhibition of RAGE antagonized S100B/RAGE over expression and sRAGE secretion in animal and astrocyte models

In addition, it was found that the usage of RAGE inhibitor FPS-ZM1 could reduce the expression of S100B, and subsequently attenuated RAGE expression and sRAGE secretion in TBI rat model (Fig. 4B, E) and astrocyte stretch injury (Fig. 4C). In addition, the administration of RAGE inhibitor FPS-ZM1 could reduce the exogenous S100B-induced increase of S100B expression and secretion in astrocytes (Additional file 1: Fig. S1). The data in Fig. 4 indicate that the release of S100B from astrocytes could promote RAGE expression and sRAGE release after TBI. The enhancement of RAGE, in turn, may have induced further up-regulation of S100B.

### The activation of S100B/RAGE signal induced endothelial glycocalyx shedding

#### Syndecan-1 level was reduced in brain tissue and increased in serum

**Fig. 5:**
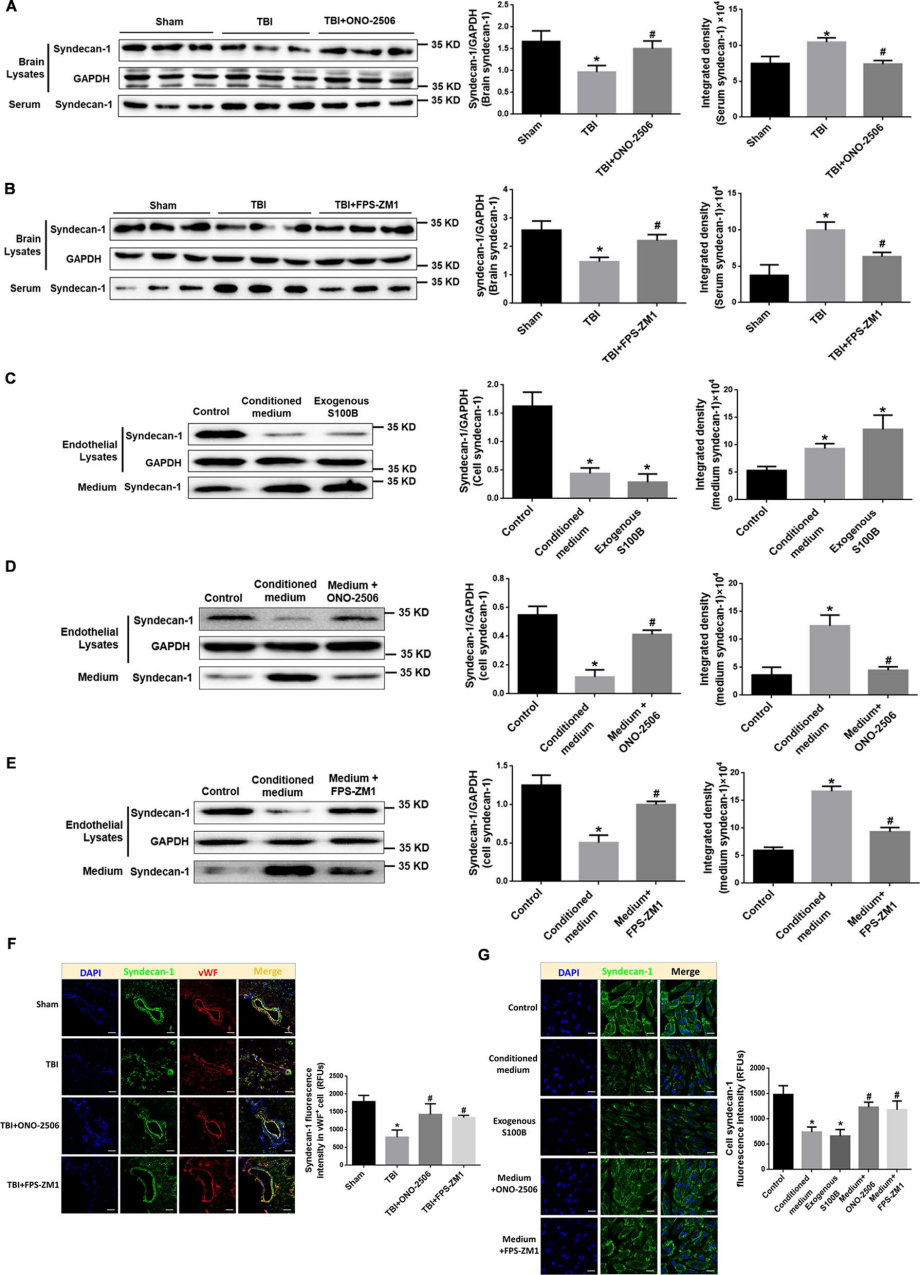
Activation of S100B/RAGE signal mediates EG damage. **A**, **B** Representative blots of syndecan-1 in injured brain and in serum of TBI rats, and TBI plus ONO-2506 (**A**) or FPS-ZM-1 (**B**). GADPH was used as a soluble loading control (left panel). In addition, quantification (histograms of right panel) of syndecan-1 in brain lysates and serum from representative blots shown in left panel. *p < 0.05 compared with Sham group, ^#^*p* < 0.05 compared with TBI group, *n* = 6. **C** Representative blots of Syndecan-1 in RAECs and in cultured medium after stretch injured-conditioned medium or exogenous S100B administration. GADPH was used as a soluble loading control (left panel). In addition, quantification (histograms of right panels) of Syndecan-1 from representative blots shown in left panel. *p < 0.05 compared with Control group, *n* = 3. **D**, **E** Representative blots of syndecan-1 in RAECs and in cultured medium after treatment of stretch injured-conditioned medium or injured medium plus S100B inhibitor ONO-2506 (**E**) or FPS-ZM-1 (**F**). GADPH was used as a soluble loading control (left panel). In addition, quantification (histograms of right panel) of syndecan-1 in cell lysates and medium from representative blots shown in left panel. *p < 0.05 compared with control group, ^#^*p* < 0.05 compared with conditioned medium-treated group, *n* = 3. **F** Representative confocal images showing the subcellular localization of syndecan-1 and vWF in the brain tissue of rats treated with TBI, TBI plus ONO-2506 or FPS-ZM-1. Scale bar = 100 μm. The immunofluorescence intensity of syndecan-1 in brain tissue from five fields per sample in each group. The relative fluorescence intensity was quantified and reported as relative fluorescence units (RFUs). *p < 0.05 compared with Sham group, ^#^*p* < 0.05 compared with TBI group, *n* = 3. **G** Representative confocal images showing the expression and distribution of syndecan-1 in RAECs treated with ONO-2506 or FPS-ZM-1. Scale bar = 50 μm. The immunofluorescence intensity of syndecan-1 in cells from five fields per sample in each group was quantified and reported as relative fluorescence units (RFUs). *p < 0.05 compared with control group, ^#^*p* < 0.05 compared with conditioned medium-treated group, *n* = 3

The results further demonstrated that the amount of syndecan-1, the glycocalyx damage markers, was decreased in brain tissue from peri-injury cortex, while serum level of syndecan-1 was increased (Fig. 5A, B). Our data revealed similar changes of syndecan-1 in a time-dependent pattern (Additional file 1: Fig. S2).

#### Inhibition of S100B/RAGE signal reduces EG damage in TBI rats

Consistently, the results from TBI rats treated with ONO-2506 or FPS-ZM1 showed a significant improvement of EG integrity with syndecan-1 more in brain tissue and less in the serum (Fig. 5A, B). The localization of syndecan-1 on the intima of micro vessels was mostly preserved by the application of ONO-2506 or FPS-ZM1 (Fig. 5F).

#### The EG damage in injured RAECs was also attenuated by inhibition of S100B/RAGE signal

The results from RAECs stimulated with conditioned medium of stretch injury astrocytes or exogenous S100B revealed that the protein level of syndecan-1 was significantly lowered in the cells and elevated in cultured medium (Fig. 5C). The changes of syndecan-1 in conditioned medium-treated group were attenuated by S100B inhibitor ONO-2506 (Fig. 5D) or RAGE inhibitor FPS-ZM1, respectively (Fig. 5E). The immunostaining of syndecan-1 in RAECs confirmed these results (Fig. 5G). The data from Fig. 5 prove that the activation of S100B/RAGE signaling mediates EG damage after TBI.

### The up-regulation of ADAM17 after TBI contributes to EG damage

#### The up-regulation of ADAM17 mediate EG damage in TBI rats

**Fig. 6:**
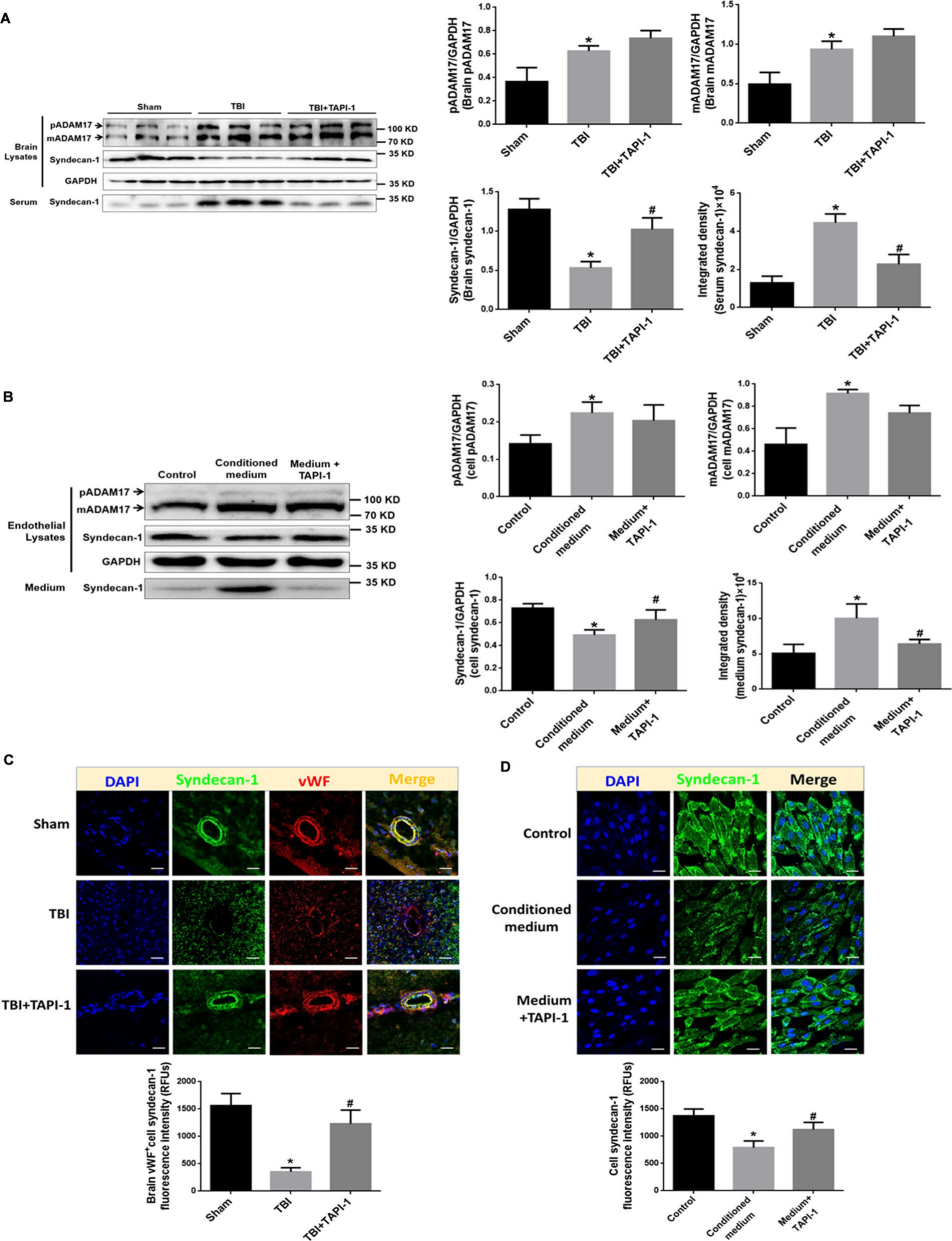
Up-regulation of ADAM17 after TBI contributes to EG damage. **A** Representative blots of pADAM17, mADAM17 and syndecan-1 in injured brain and in serum of TBI rats, and TBI plus TAPI-1. GADPH was used as a soluble loading control (left panel). In addition, quantification (histograms of right panels) of pADAM17, mADAM17, and syndecan-1 in brain lysates and in serum from representative blots shown in left panel. *p < 0.05 compared with Sham group, ^#^*p* < 0.05 compared with TBI group, *n* = 6. **B** Representative blots of pADAM17, mADAM17 and syndecan-1 in RAECs and in cultured medium after treatment of stretch injured-conditioned medium or injured medium plus ADAM17 inhibitor TAPI-1. GADPH was used as a soluble loading control (left panel). In addition, quantification (histograms of right panels) of pADAM17, mADAM17, and syndecan-1 in cell lysates and in medium from representative blots shown in left panel. *p < 0.05 compared with control group, ^#^*p* < 0.05 compared with conditioned medium-treated group, *n* = 3. **C** Representative confocal images showing the subcellular localization of syndecan-1 and vWF in the brain tissue of TBI rats treated with TAPI-1. Scale bar = 100 μm. The immunofluorescence intensity of syndecan-1 in brain tissue from five fields per rat in each group. The relative fluorescence intensity was quantified and reported as relative fluorescence units (RFUs). *p < 0.05 compared with Sham group, ^#^*p* < 0.05 compared with TBI group, *n* = 3. **D** Representative confocal images showing the expression and distribution of syndecan-1 in RAECs treated with TAPI-1. Scale bar = 50 μm. The immunofluorescence intensity of syndecan-1 in cells from five fields per sample in each group was quantified and reported as relative fluorescence units (RFUs). *p < 0.05 compared with control group, ^#^*p* < 0.05 compared with conditioned medium-treated group, *n* = 3

The results from TBI rats revealed that the protein levels of pADAM17 (the immature form) and mADAM17 (the mature form) from peri-injury cortex were both higher than those in sham group, along with the increase of syndecan-1 in serum, while the application of ADAM17 inhibitor TAPI-1, a specific hydroxamate inhibitor of metalloprotease disintegrins, effectively blocked this TBI-induced syndecan-1 shedding (Fig. 6A). Similar results were obtained under confocal immunofluorescent, and TAPI-1 specifically preserved the localization of syndecan-1 on the intima of microvessels (Fig. 6C).

#### The up-regulation of ADAM17 mediate EG damage in RAECs stimulated by conditioned medium

The results from RAECs cultured in conditioned medium consistently demonstrated that the protein levels of pADAM17 (the immature form) and mADAM17 (the mature form) were both higher than those in control or sham group, accompanying with the increased release of syndecan-1 in the medium, thus indicating the EG shedding. This induced syndecan-1 shedding was effectively blocked by ADAM17 inhibitor, TAPI-1, with no significant change in pADAM17 and mADAM17 protein levels (Fig. 6B). The immunostaining of syndecan-1 in RAECs confirmed these results (Fig. 6D). These data in Fig. 6 indicate that enhanced expression and the activation of ADAM17 might contribute to the shedding of syndecan-1 and TAPI-1 might be effective on the preservation of glycocalyx after TBI.

### Activating S100B/RAGE signaling promotes ADAM17 expression and activation after TBI

#### Inhibition of S100B and RAGE expression reduces ADAM17 expression and activation in TBI rats

**Fig. 7:**
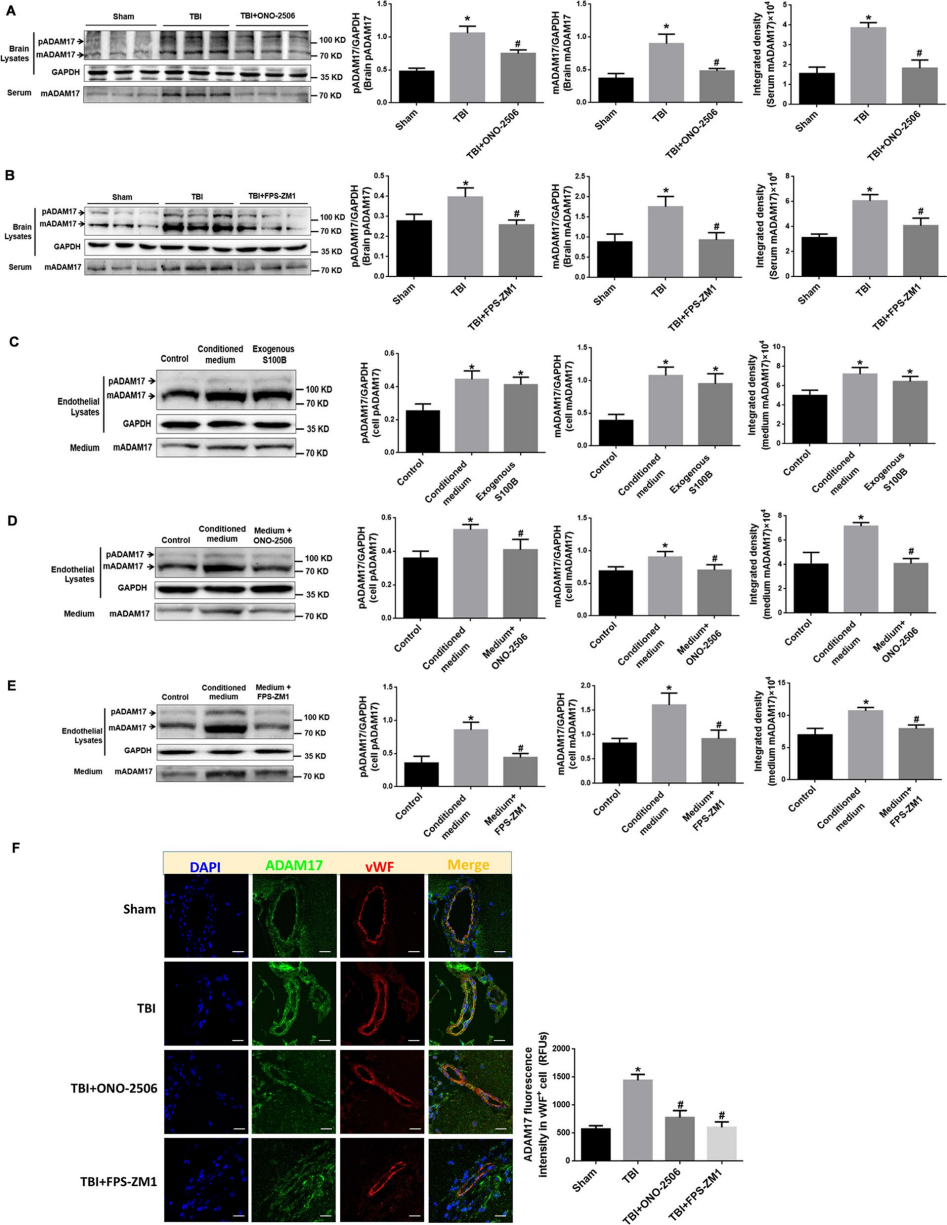
Activating S100B/RAGE signaling promotes ADAM17 expression and activation after TBI. **A**, **B** Representative blots of pADAM17, mADAM17 in injured brain and mADAM17 in serum of TBI rats, and TBI plus ONO-2506 (**A**) or FPS-ZM-1 (**B**). GADPH was used as a soluble loading control (left panel). In addition, quantification (histograms of right panel) of pADAM17, mADAM17 in brain lysates and mADAM17 in serum from representative blots shown in left panel. *p < 0.05 compared with Sham group, ^#^*p* < 0.05 compared with TBI group, *n* = 6. **C** Representative blots of pADAM17, mADAM17 in RAECs and mADAM17 in cultured medium after stretch injured-conditioned medium or exogenous S100B administration. GADPH was used as a soluble loading control (left panel). In addition, quantification (histograms of right panel) of pADAM17, mADAM17 from representative blots shown in left panel. *p < 0.05 compared with Control group, *n* = 3. **D**, **E** Representative blots of pADAM17, mADAM17 in RAECs and mADAM17 in cultured medium after treatment of stretch injured-conditioned medium or injured medium plus S100B inhibitor ONO-2506 (**D**) or FPS-ZM-1 (**E**). GADPH was used as a soluble loading control (left panel). In addition, quantification (histograms of right panel) of pADAM17, mADAM17 in cell lysates and medium from representative blots shown in left panel. *p < 0.05 compared with Control group, ^#^*p* < 0.05 compared with conditioned medium-treated group, *n* = 3. **F** Representative confocal images showing the subcellular localization of ADAM17 with vWF in the brain tissue of rats treated with TBI, TBI plus ONO-2506 or FPS-ZM-1. Scale bar = 50 μm. The immunofluorescence intensity of ADAM17 in brain tissue from five fields per sample in each group were quantified and reported as relative fluorescence units (RFUs). *p < 0.05 compared with Sham group, ^#^*p* < 0.05 compared with TBI group, *n* = 3

While the above-mentioned results have proved that the activation of S100B/RAGE signaling mediates EG damage, it is worthwhile to elucidate whether S100B/RAGE signal could have been involved in the regulation of ADAM17 after TBI. The protein levels of pADAM17 and mADAM17 were both significantly elevated in brain tissue and mADAM17 level was also increased in serum from TBI rats, indicating the secretion of mADAM17 from cells. The administration of S100B or RAGE inhibitor abolished TBI-induced up-regulation of pADAM17 and mADAM17 in brain tissue, and attenuated the secretion of mADAM17 into serum (Fig. 7A, B). The immunostaining of injury-adjacent brain tissue revealed a significant increased co-localization of ADAM17 with endothelial marker vWF and the inhibition of S100B or RAGE attenuated this increase (Fig. 7F).

#### The increased S100B and RAGE expression promotes ADAM17 expression and activation in RAECs

The results from RAECs also revealed that the protein levels of pADAM17 and mADAM17 were significantly elevated in RAECs cultured with exogenous S100B. It is interesting to note that mADAM17 was also elevated in RAEC medium, indicting the secretion of mADAM17 from endothelial cells (Fig. 7C). Compared to conditioned medium-treated alone, the addition of S100B inhibitor ONO-2506 (Fig. 7D) or RAGE inhibitor FPS-ZM1 (Fig. 7E) exhibited a significant reduction in pADAM17 and mADAM17 expression and secretion of mADAM17. These data from Fig. 7 imply that activating S100B/RAGE signaling may promote ADAM17 expression and activation.

### Activating S100B/RAGE signal promotes ADAM17 translocation in RAECs

**Fig. 8:**
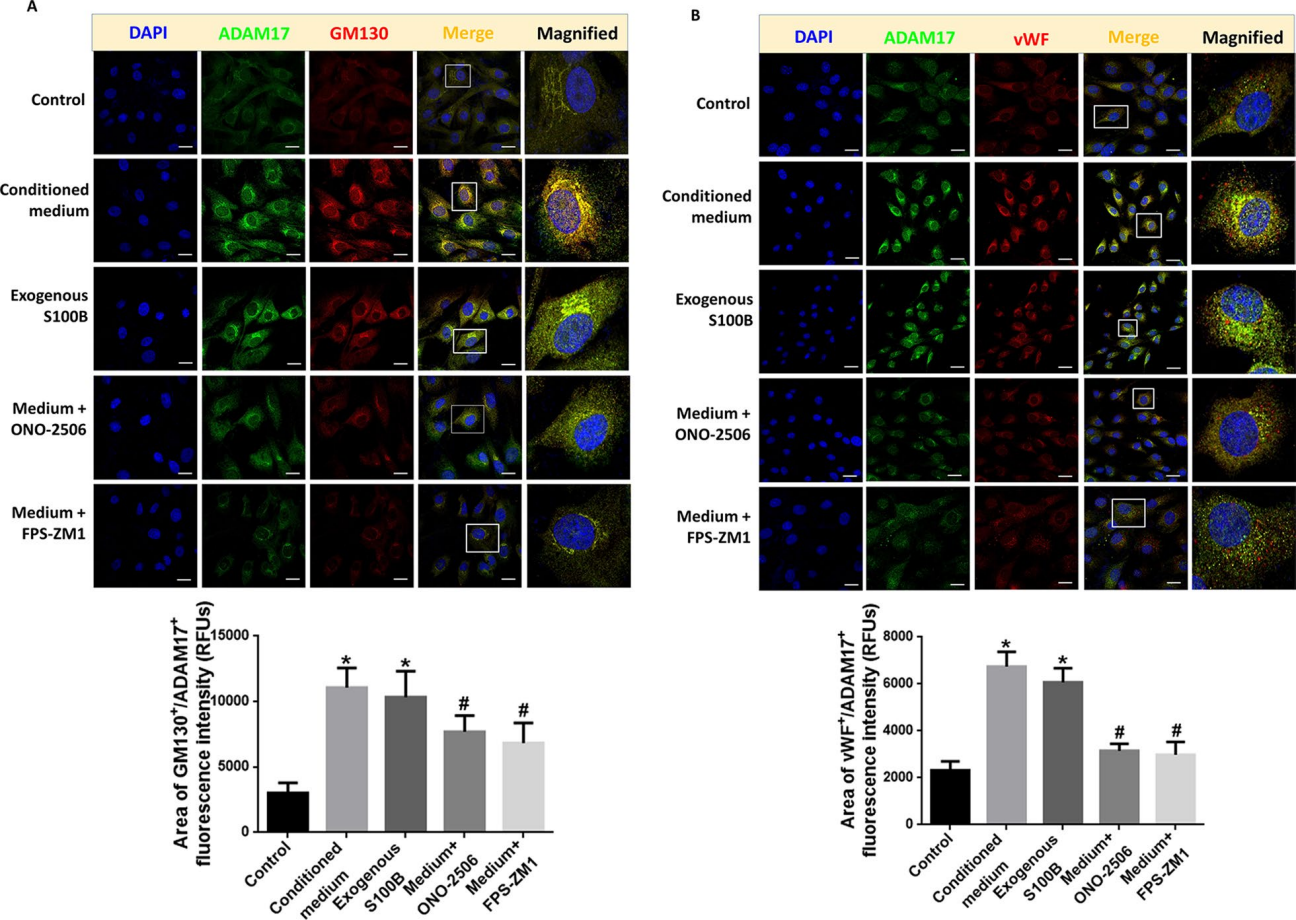
Activating S100B/RAGE signal promotes ADAM17 translocation in RAECs. **A** Representative confocal images showing the subcellular localization of ADAM17 and GM130 (the Golgi complex-associated protein) in RAECs treated with stretch injured-conditioned medium, exogenous S100B, and injured medium plus S100B inhibitor ONO-2506 or RAGE inhibitor FPS-ZM-1. Scale bar = 20 μm. The immunofluorescence intensity of GM130^+^/ADAM17^+^ in cell from five fields per sample in each group was quantified and reported as relative fluorescence units (RFUs). *p < 0.05 compared with control group, ^#^*p* < 0.05 compared with conditioned medium-treated group, *n* = 3. **B** Representative confocal images showing the subcellular localization of ADAM17 and vWF in RAECs treated with stretch injured-conditioned medium, exogenous S100B, and injured medium plus ONO-2506 or FPS-ZM-1. Scale bar = 20 μm. The immunofluorescence intensity of vWF^+^/ADAM17^+^ in cells from five fields per sample in each group was quantified and reported as relative fluorescence units (RFUs). *p < 0.05 compared with control group, ^#^*p* < 0.05 compared with conditioned medium-treated group, *n* = 3

To further verify whether S100B/RAGE signal promotes ADAM17 maturation and processing in the Golgi apparatus, the cellular location of ADAM17 and GM130, a Golgi complex-associated protein, was determined by confocal immunofluorescent. The results showed that the intensity of ADAM17 colocalized with GM130 in RAECs was obviously increased after treatment of conditioned medium and exogenous S100B. The application of S100B inhibitor ONO-2506 or RAGE inhibitor FPS-ZM1 significantly attenuated this increased colocation (Fig. 8A). The results of double immunostaining of ADAM17 and vWF further verified that the increased ADAM17 was colocalized with vWF much more than control after conditioned medium and exogenous S100B treatment. In addition, the administration of S100B or RAGE inhibitor significantly attenuated this co-localization with separated staining of ADAM17 and vWF, in RAECs (Fig. 8B). These results suggest that ADAM17 could be secreted out of endothelial cells through activation of vWF-rich W–P bodies.

### S100B/RAGE-mediated up-regulation of ADAM17 promotes secondary injury after TBI

**Fig. 9:**
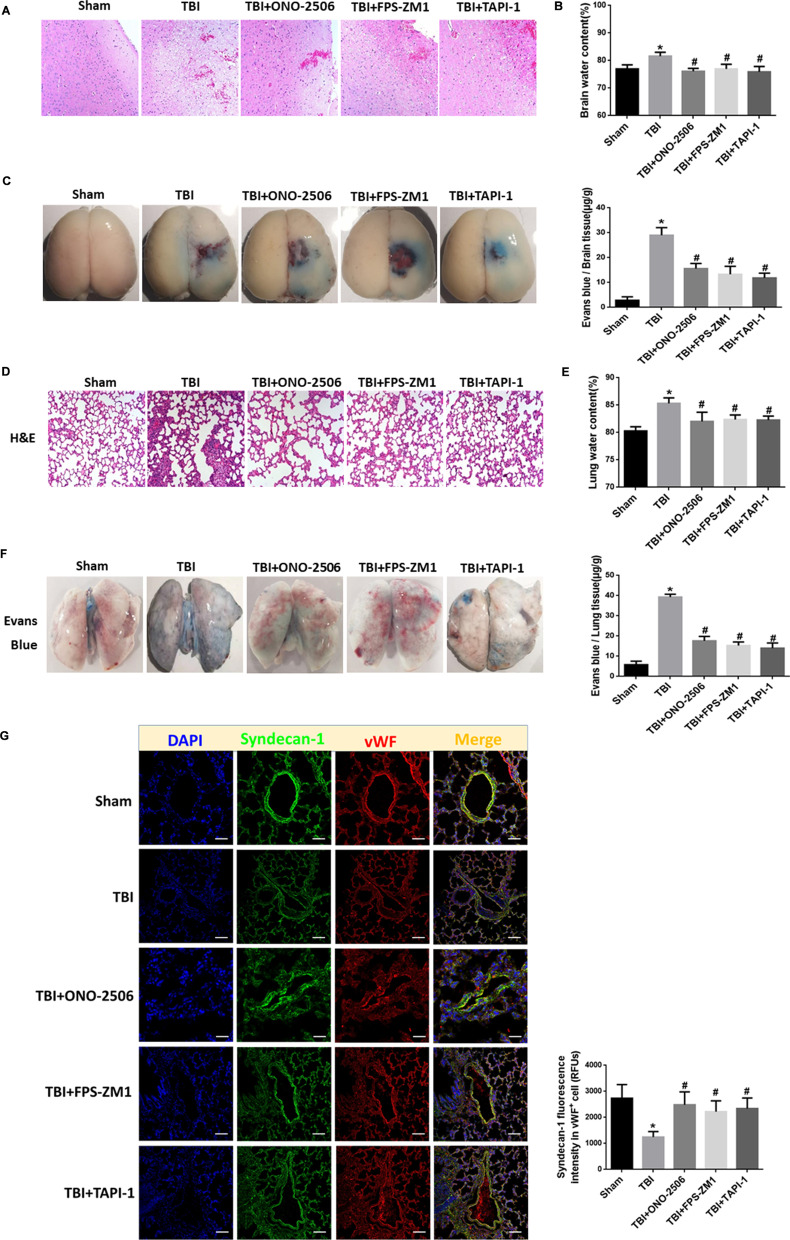
S100B/RAGE mediated activation of ADAM17 promotes secondary brain and lung injury after TBI. **A**, **D** Representative H&E staining images showing the histology of cortex of injured-side brain tissue (**A**) or lungs (**D**) of rats 6 h after the onset of TBI in TBI, and TBI plus S100B inhibitor ONO-2506, RAGE inhibitor FPS-ZM-1, or ADAM17 inhibitor TAPI-1 group, (×100). **B**, **E** Quantification of water content of brain (**B**) or lungs (**E**) from rats of TBI and TBI plus respective inhibitor. *p < 0.05 compared with Sham group; ^#^*p* < 0.05 compared with TBI group, *n* = 6. **C**, **F** Gross observation of brains (**C**) and lungs (**F**) with Evans blue indicating the vascular leakage of tissue in rats of TBI and TBI plus respective inhibitor. In addition, quantification of Evans blue concentration in tissue from rats of TBI, and TBI plus respective inhibitor (far right histogram). *p < 0.05 compared with Sham group; ^#^*p* < 0.05 compared with TBI group, *n* = 6. **G** Representative confocal images showing the subcellular localization of syndecan-1 and vWF in lung tissue from rats 6 h after the onset of TBI, and TBI plus S100B inhibitor ONO-2506, RAGE inhibitor FPS-ZM-1, or ADAM17 inhibitor TAPI-1 group. Scale bar = 100 μm. The immunofluorescence intensity of syndecan-1 in lung tissue from five fields per rat in each group. The relative fluorescence intensity was quantified and reported as relative fluorescence units (RFUs). *p < 0.05 compared with Sham group, ^#^*p* < 0.05 compared with TBI group, *n* = 3

The results from TBI rats demonstrated that the administration of S100B, RAGE, and ADAM17 inhibitors, e.g., ONO-2506, FPS-ZM1, or TAPI-1 could, respectively, attenuated injury-induced histopathological damage (Fig. 9A), vascular leakage (Fig. 9C) in the surrounding tissue of the brain and reduced TBI-induced brain edema (Fig. 9B), and subsequently, reduced the mortality rate of TBI (Table 2). These results clarify the role of S100B/RAGE mediated ADAM17 signaling in secondary brain injury after TBI.

The remote effect of TBI on lungs was confirmed by glycocalyx shedding on intima of pulmonary vessels (Fig. 9D), histopathological damage (Fig. 9E), increased vascular leakage (Fig. 9F) and water content (Fig. 9G), in the lungs. The administration of inhibitors ONO-2506, FPS-ZM1, or TAPI-1 could, respectively, protect against the secondary lung injury after TBI (Fig. 9D–G). These data indicate that secreted ADAM17 from endothelial cells could reach distant organs through circulation and S100B/RAGE-mediated up-regulation of ADAM17 might promote secondary lung injury after TBI.

## Discussion

The present study demonstrated that in both animal and cellular model, TBI triggered the elevation of S100B in the brain tissue and serum. The release of S100B might work with RAGE through paracrine and systemic response, resulting in further synthesis and secretion of S100B from astrocytes. The activation of S100B/RAGE signal after TBI could mediate endothelial glycocalyx shedding by enhancing the protein expression and enzyme activity of ADAM17 in endothelial cells. We further confirmed that the activation of S100B/RAGE could also induce the Golgi translocation of ADAM17 and its localization in W–P bodies of RAECs, subsequently promoting endothelial glycocalyx damage, and aggravating brain tissue injury. Systemically, the activation of S100B/RAGE–ADMA17 pathway could cause the damage of pulmonary microvascular endothelial glycocalyx, increased vascular permeability, and consequently leading to secondary lung injury.

### TBI triggers the release of S100B

S100B has been considered as a biomarker of brain injury and the increase of S100B in serum can reflect the early changes of BBB function and nerve cell damage [26, 27]. Consistent with many clinical observations, in present study, the results from TBI models in vivo by experimental lateral fluid percussion injury in rats and in vitro by stretch injury in primary astrocytes demonstrated significant increases in S100B level both in brain and serum, but also and in astrocytes and medium early after injury. Several studies have shown that the overproduction of S100B by activated astrocytes after brain injury further enhances microglial and astrocyte activation, leading to neuroinflammation [7, 28]. Our study also found that inhibition of S100B could significantly alleviate the pathological damage of brain and lung tissues, indicating that S100B plays an important role in mediating the secondary injury of TBI by working as one of the DAMPs [29].

### S100B and RAGE mutually regulate their expression and activation

S100B could exert its effects by binding with RAGE [30]. In our study, the level of RAGE expression in brain tissue and astrocytes, and the level of sRAGE in serum and medium were all significantly up-regulated by TBI or stretch injury. The inhibition of S100B attenuated those up-regulation and the inhibition of RAGE reversed the enhancement of S100B either, indicating the mutual regulation of S100B and RAGE. The inhibition of RAGE also attenuated TBI-induced brain and lung damage, and improved astrocyte viability after stretch injury, suggesting that the activation of the ligand/RAGE signaling pathway may be an important factor in mediating the secondary damage of TBI.

### The activation of S100B/RAGE results in endothelial glycocalyx shedding

Current studies have shown that the integrity of endothelial glycocalyx is damaged to varying degrees in TBI, especially in secondary injury [31, 32]. Indicating by the changes of syndecan-1 content and location in brain tissue and serum, as well as in endothelial and cultured medium, the results in present study demonstrated that astrocyte-derived S100B and the subsequent RAGE activation on endothelial cells after TBI and stretch injury can induce endothelial glycocalyx damage in brain and lung as well. These data suggested the S100B/RAGE-induced glycocalyx shedding from endothelial cells might be one of the critical steps in initiating secondary damage, such as BBB dysfunction, brain edema or remote organ injury after TBI.

### S100B/RAGE evokes EG damage by inducing ADAM17 expression and translocation

For the mechanism of S100B/RAGE-induced glycocalyx shedding, ADAM17, one of the major sheddases, emerged in our study. ADAM17 exists as immature proform (pADAM17) and as mature protease (mADAM17) in cells [33, 34]. In this study, we first demonstrated that the changes of syndecan-1 after TBI was accompanied with significant increased expression of both pADAM17 and mADAM17, implying the involvement of ADAM17 expression in endothelial glycocalyx shedding during the development of secondary TBI. A variety of inflammatory mediators are responsible in stimulating ADAM17 expression [33]. For the first time, present study found that the application of exogenous S100B enhanced the expression of pADAM17 and mADAM17, and the inhibition of S100B/RAGE signaling abolished TBI-induced ADAM17 expression, suggesting the activation of S100B/RAGE pathway triggered the expression of ADAM17 after TBI.

The sheddase activity of ADAM17 is modulated by the translocation of ADAM17 from ER to Golgi apparatus, where the maturation takes place [35]. This study found that the inhibition of S100B/RAGE signaling abolished the increase location of ADAM17 in Golgi apparatus after TBI, confirming that TBI-induced activation of S100B/RAGE pathway also regulates the translocation and maturation of ADAM17. Most of the mature ADAM17 seems to be intracellularly located, while only a small amount is actually at the cell surface, where shedding can take place [33, 36]. The S100B/RAGE-enhanced localization of mADAM17 with vWF in RAEC W–P bodies indicated that ADAM17 could be secreted through the release of W–P bodies from injured endothelial cells, then reach to distant organs and tissue [37]. Our findings imply that the detection of plasma ADAM17 level and activity might help to monitor disease progression in TBI with endothelial involvement [38].

### The inhibition of ADAM17 activity is capable of attenuating EG damage

The activation of mADAM17 relied on trafficking to the cell surface, where shedding can take place [34]. In this study, ADAM17 inhibitor, TAPI-1, a specific hydroxamate inhibitor of metalloprotease disintegrins, reversed the elevation of syndecan-1, without changing the content of pADAM17 and mADAM17, confirming the activation of ADAM17 after maturation in inducing glycocalyx shedding. The impaired glycocalyx barrier is more conducive to inflammatory adhesion to endothelial cells, promoting thrombosis and cell damage, thus leading to a vicious circle [10]. In this study, the inhibition of ADAM17 activity with TAPI-1 could significantly reverse the shedding of endothelial glycocalyx both in endothelial cells and brain tissue, implying the effect of antagonizing ADAM activity in protecting endothelial barrier structure.

### The S100B/RAGE–ADMA17-induced EG damage is involved in primary and secondary TBI

Astrocytes and endothelial cells are the key cells to maintain the normal function of BBB [39]. In this study, the protective effect of inhibiting S100B/RAGE signaling and ADAM17 activation on pathological injury and BBB dysfunction in TBI rat model confirms the involvement of this S100B/RAGE-induced astrocyte activation and ADAM17-evoked endothelial glycocalyx shedding in the development of primary and secondary TBI in vivo*.*

TBI-induced acute lung injury (TBI-induced ALI) is regarded as the most common complication of severe TBI that is an independent predictor of poor outcomes in TBI patients and strongly increases the mortality [40]. Concurred with those seen in other studies [41, 42], our previous study has shown that TBI-induced activation of S100B could mediated the development of neutrophil extracellular traps (NETs) in the lung, leading to subsequent ALI [43]. Present study provides more evidence to reveal that S100B/RAGE and ADAM17 activation triggered the shedding of pulmonary endothelial glycocalyx and this change could be rescued with inhibitors targeting this pathway, suggesting that S100B/RAGE/ADAM17 is not only a key signal of astrocyte and endothelial cell damage, but also an important participant in local or systemic secondary injury after TBI. Therefore, we should actively remedy the complications such as lung injury while treating the primary injury, and prevent the secondary injury from further aggravating the pathogenetic condition of TBI patients.

There are some limitations to consider when drawing conclusions from this study. First, the activation of ADAM17 is a multi-step, gradually progressed and strictly regulated process, the critical regulatory mechanism of S100B/RAGE signal on this complex process remains to be further studied. Second, while the secondary brain and lung injuries cause the major organ dysfunctions after TBI, the effect of S100B/RAGE/ADAM17 pathway on other organ functions, such as coagulation and renal function, remains to be investigated. Third, pharmacological interventions used in study still have some unavoidable shortcomings, further RNA interference and gene knocking out should be considered in future research.

## Conclusions

**Fig. 10:**
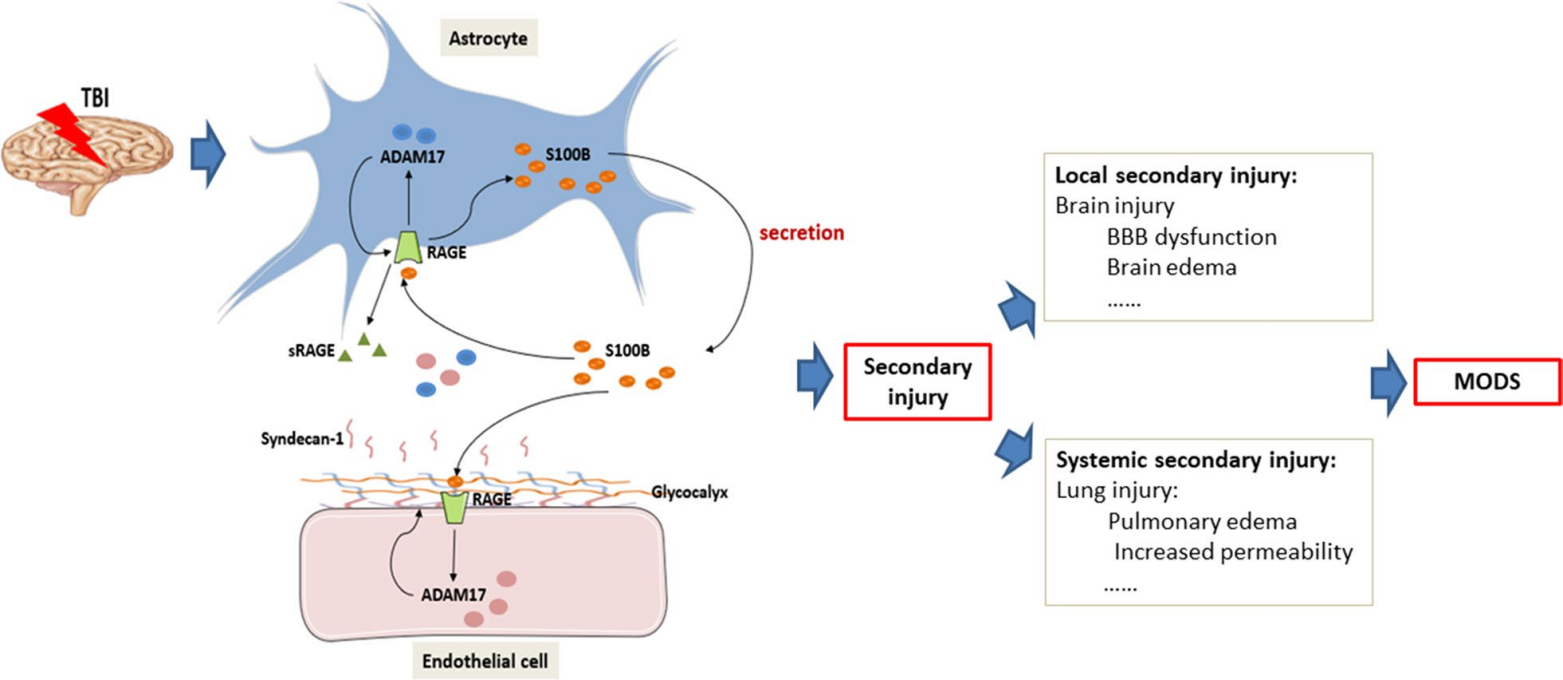
Schematic image shows the involvement of S100B/RAGE-enhanced ADAM17 activation in the development of traumatic brain injury. At the onset of TBI, the upregulation of S100B in astrocytes are rapidly released and accumulates in extracellular space, which could exert its effect back on astrocytes through paracrine, and on endothelial cells through systemic circulation. The binding of S100B with its receptor RAGE stimulates astrocytes to further enhance the synthesis and secretion of S100B. Then, the activation of S100B/RAGE signal and its specific up-regulation on ADAM17 promotes the shedding of endothelial glycocalyx, aggravates the dysfunction of BBB, and increases the vascular permeability, leading to secondary brain and lung injury, or even multiple organ dysfunction syndromes (MODS)

TBI triggers the up-regulation of S100B in astrocytes, the latter could exert its effect back on astrocytes through paracrine, and on endothelial cells through systemic circulation by binding with RAGE. The activation of S100B/RAGE signal and its specific regulation on ADAM17 expression, translocation and activation further promotes the shedding of endothelial glycocalyx, aggravates the dysfunction of BBB, and increases the vascular permeability, leading to secondary brain and lung injury, or even multiple organ dysfunction syndromes (MODS) (Fig. 10). Although we are still far from comprehending the molecular pathophysiology of TBI-related secondary injury, present study might have a way for a deeper understanding of the molecular processes that are responsible for the vascular barrier impairment in TBI.

## Supplementary Information

**Additional file 1: Figure S1.** Representative blots of S100B in astrocytes and in cultured medium after treatment of exogenous S100B or exogenous S100B plus RAGE inhibitor FPS-ZM1. GADPH was used as a soluble loading control (left panel). In addition, quantification (histograms of right panels) of S100B in cell lysates and in medium from representative blots shown in left panel. *p < 0.05 compared with control group, ^*#*^*p* < 0.05 compared with exogenous S100B group, *n* = 3. **Figure S2.** Representative blots of syndecan-1 in tissue lysates of brain peri-injury cortex and the serum at different timepoints after the onset of TBI. GADPH was used as a soluble loading control (left panel). In addition, quantification (histograms of right panels) of syndecan-1 from representative blots shown in left panel. *p < 0.05 compared with Sham group, *n* = 6.

## Data Availability

The data sets used and/or analyzed in the current study are available from the corresponding authors on request.

## Abbreviations

TBI: Traumatic brain injury
DAMPs: Damage-associated molecular patterns
RAGE: Advance glycation endproducts
EG: Endothelial glycocalyx
ADAM17: A disintegrin and metalloprotease 17
TACE: Tumor necrosis factor (TNF)-alpha converting enzyme
GFAP: Glial fibrillary acid protein
RAECs: Rat aortic endothelial cells
vWF: Von Willebrand factor
ALI: Acute lung injury
NETs: Neutrophil extracellular traps
MODS: Multiple organ dysfunction syndromes
