# Supplementary figures and images for: The role of S100B/RAGE-enhanced ADAM17 activation in endothelial glycocalyx shedding after traumatic brain injury
Source: J Neuroinflammation. 2022 Feb 11;19:46. doi: 10.1186/s12974-022-02412-2 (PMC8832692; doi:10.1186/s12974-022-02412-2)

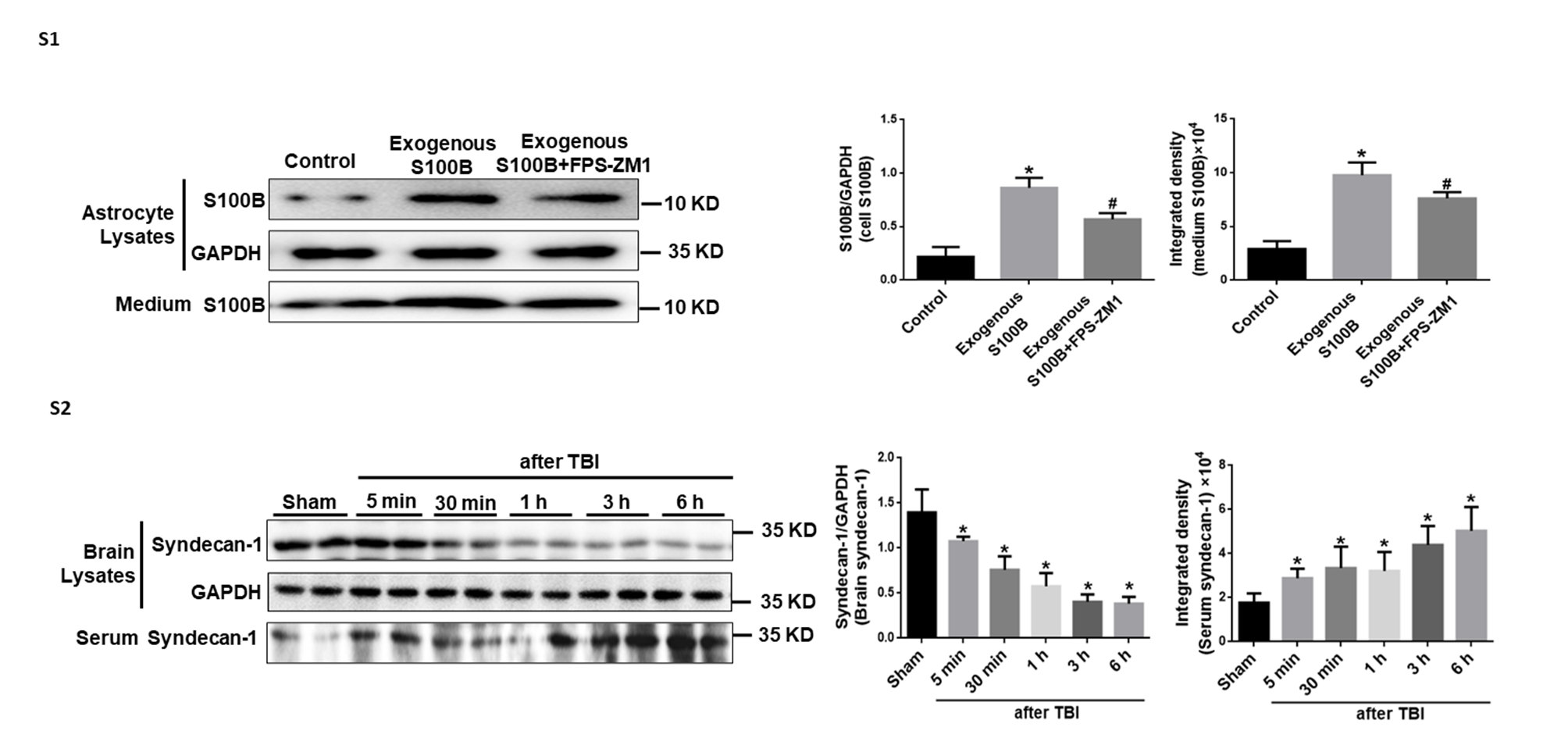

Supplement: Supplementary file 1 — Additional file 1: Figure S1. Representative blots of S100B in astrocytes and in cultured medium after treatment of exogenous S100B or exogenous S100B plus RAGE inhibitor FPS-ZM1. GADPH was used as a soluble loading control (left panel). In addition, quantification (histograms of right panels) of S100B in cell lysates and in medium from representative blots shown in left panel. *p < 0.05 compared with control group, #p < 0.05 compared with exogenous S100B group, n = 3. Figure S2. Representative blots of syndecan-1 in tissue lysates of brain peri-injury cortex and the serum at different timepoints after the onset of TBI. GADPH was used as a soluble loading control (left panel). In addition, quantification (histograms of right panels) of syndecan-1 from representative blots shown in left panel. *p < 0.05 compared with Sham group, n = 6. [file 12974_2022_2412_MOESM1_ESM.tif]
